# Supplementary figures and images for: Antibiotic Treatment Drives the Diversification of the Human Gut Resistome
Source: Genomics Proteomics Bioinformatics. 2019 Apr 23;17(1):39–51. doi: 10.1016/j.gpb.2018.12.003 (PMC6520913; doi:10.1016/j.gpb.2018.12.003)

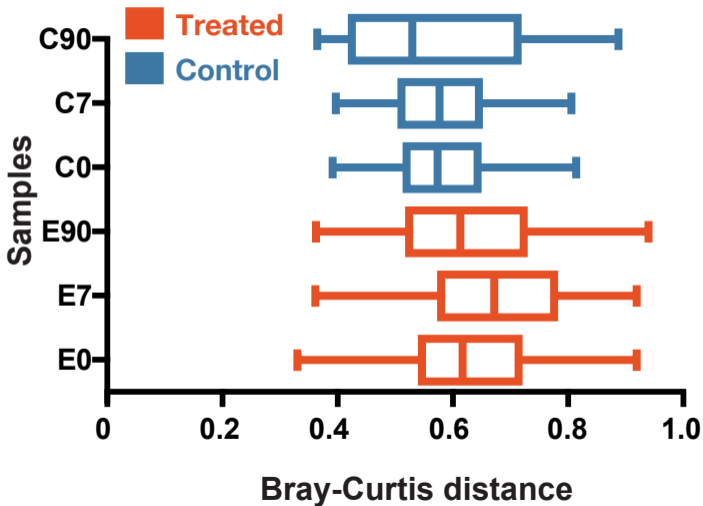

Supplement: Supplementary Figure S1 — The average Bray–Curtis distance based on the ARGs abundance profile between individuals E0, E7, and E90 refer to day 0, 7, and 90 in the treated group, respectively. C0, C7, and C90 refer to day 0, 7, and 90 in the control group, respectively. [file mmc1.pdf]

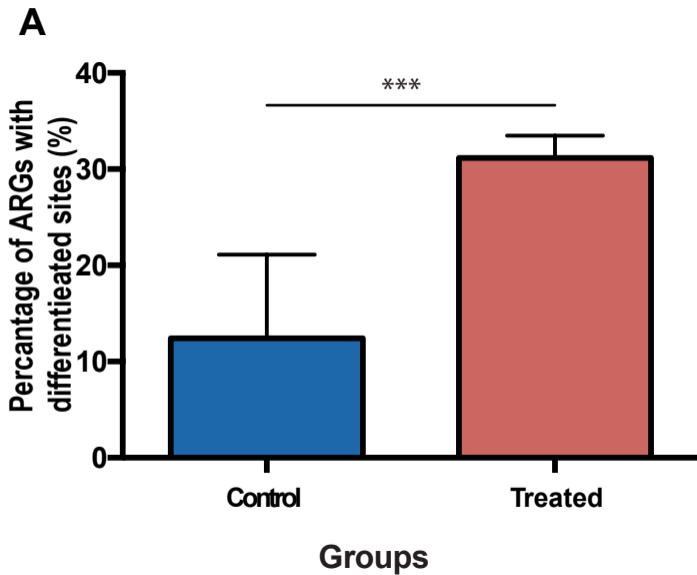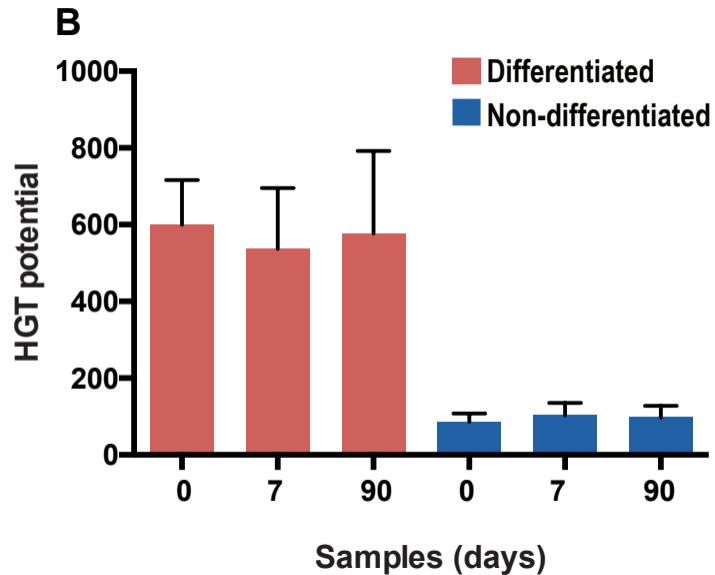

Supplement: Supplementary Figure S2 — Variation of the HGT potential over time for two types of ARGs in the control subjects [file mmc2.pdf]

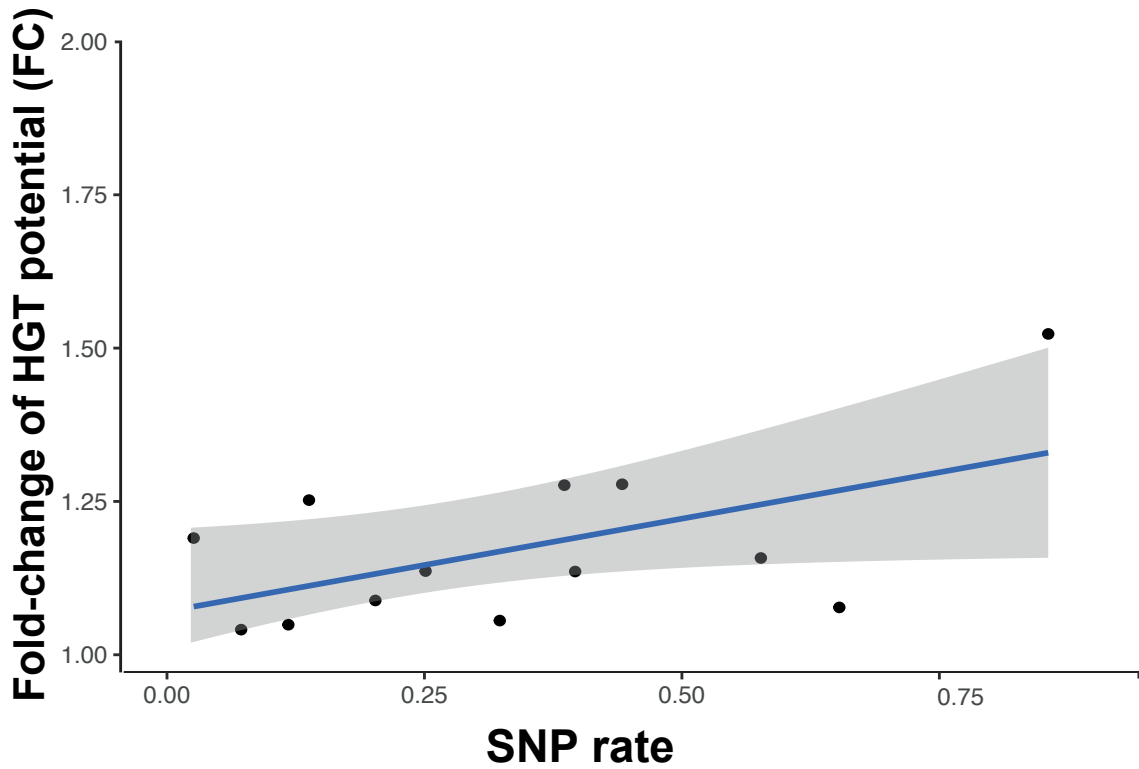

Supplement: Supplementary Figure S3 — Correlation between the variation of HGT potentials and SNP rate Pearson’s correlation (correlation coefficient 0.50 with P value = 0.038) between the per sample gene-level change of HGT potential and the gene SNP rate (proportions of differentiated sites). The ribbons reflect the standard error of the regression. [file mmc3.pdf]

*Ruminococcus brommii* 62047

A

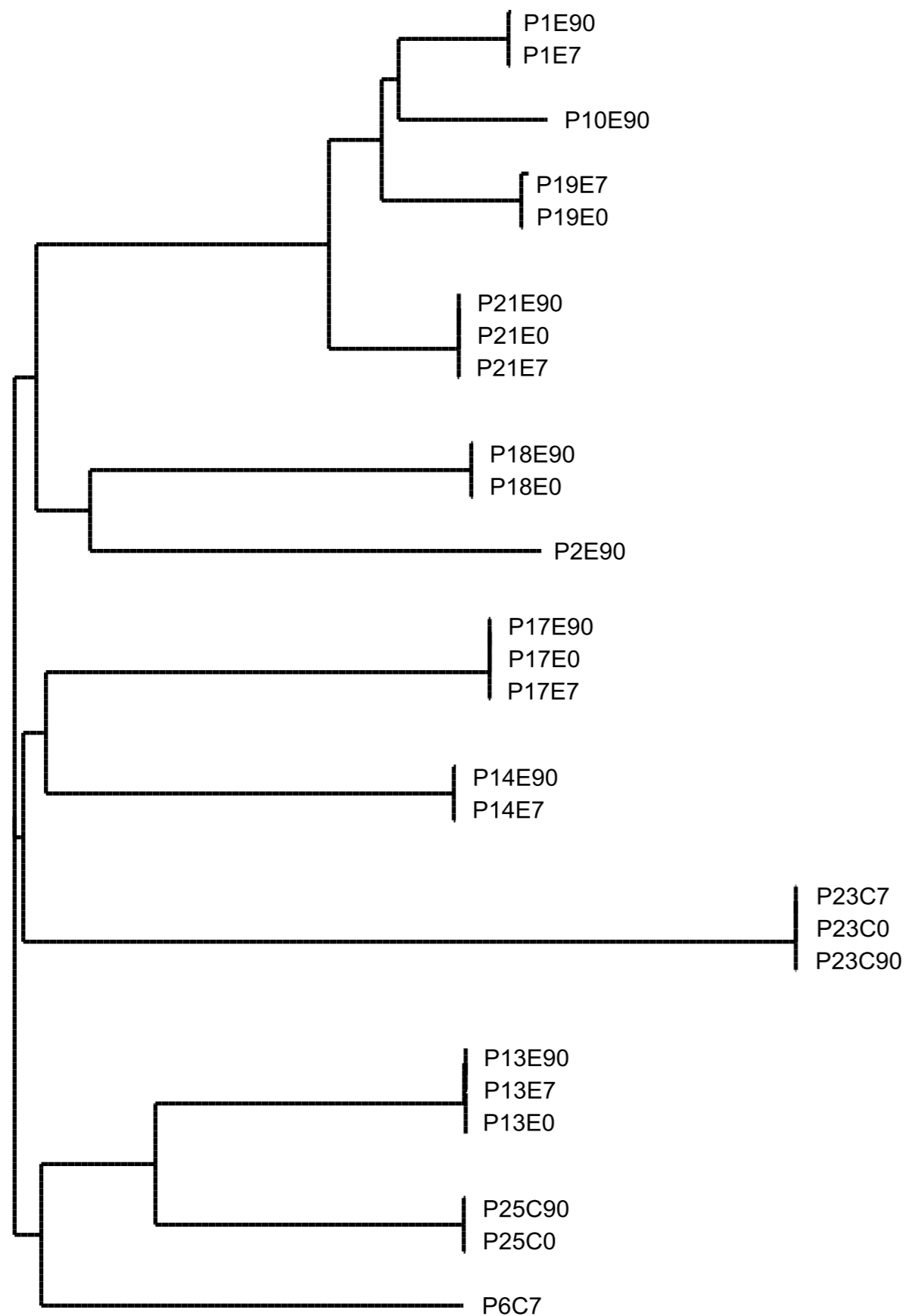

B

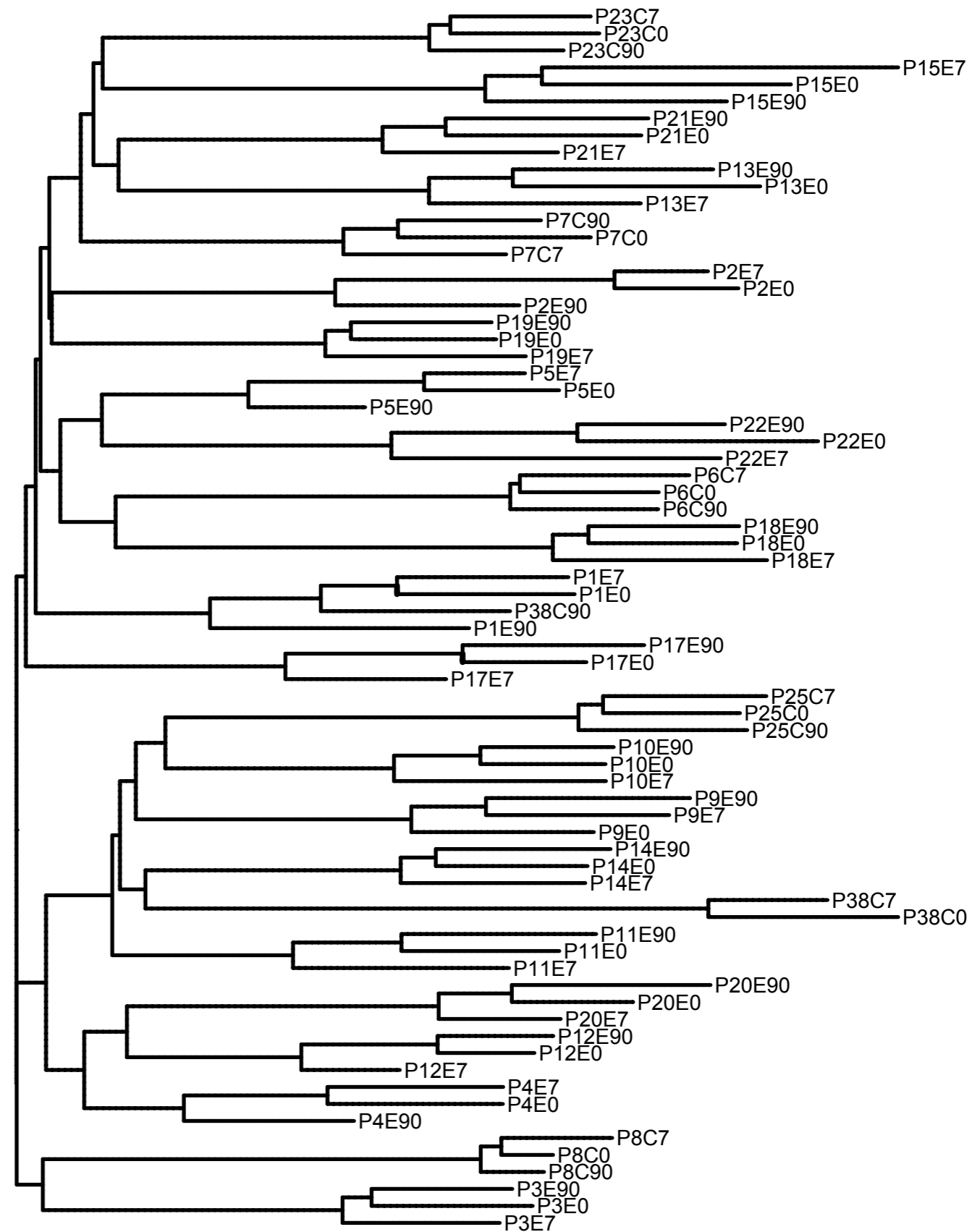

Supplement: Supplementary Figure S4 — The phylogenomic tree of A. dominant strain in species Ruminococcus bromii and B. all dominant genotypes of antibiotic resistance genes [file mmc4.pdf]

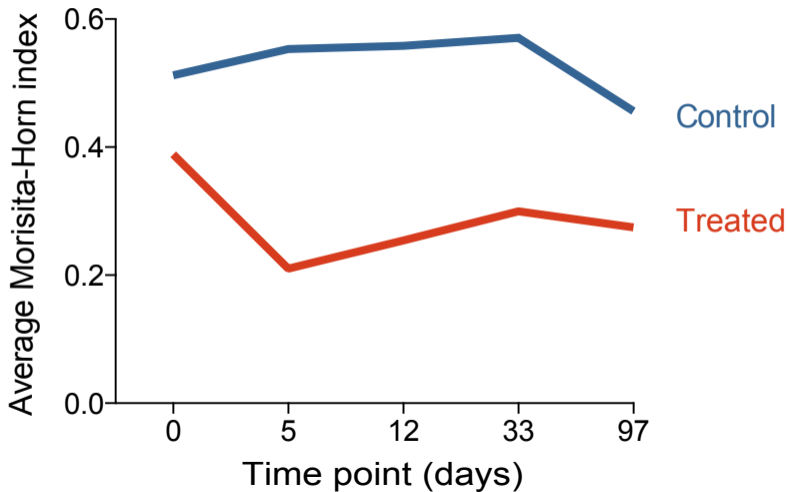

Supplement: Supplementary Figure S5 — The average Morisita–Horn dissimilarity of the resistant and controlled communities cultured [file mmc5.pdf]

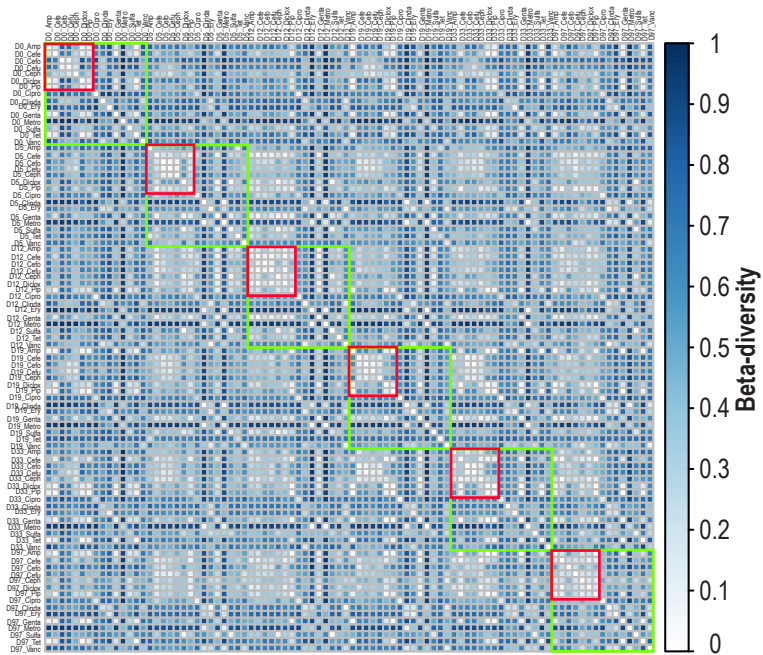

Supplement: Supplementary Figure S6 — The pairwise Morisita–Horn dissimilarity (beta-diversity) between cultured communities with antibiotics from all time points Green squares surround all pairwise community dissimilarity between all plates at each time point. Red squares surround all pairwise community dissimilarity between beta-lactam plates at each time point. Cefo, cefotaxime; Ceph, cephalexin; Cefu, cefuroxime; Pip, pipercillin; Cefe, cefepime; Amp, ampicillin; Diclo, Dicloxacillin; Cipro, Ciprofloxacin; Sulfa, Sulfamethoxazole; Tet, Tetracycline; Clinda, Clindamycin; Ery, Erythromycin; Metro, Metronidazole; Vanc, Vancomycin. [file mmc6.pdf]

Nucleotide diversity

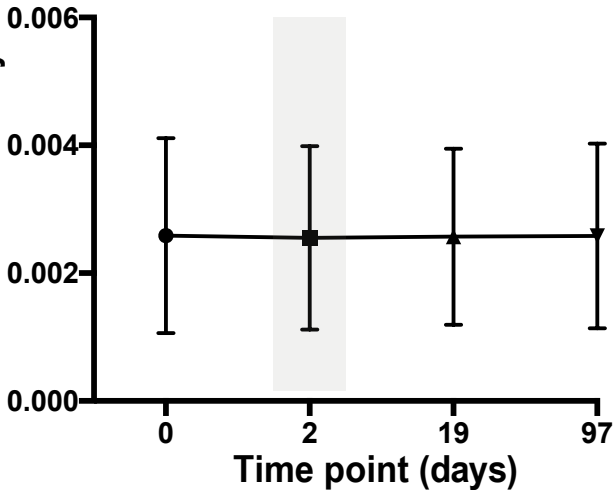

Supplement: Supplementary Figure S7 — Variation of the genome-wide nucleotide diversity over time in the control subject The nucleotide diversity reflects the average number of nucleotide differences per site between sequences. [file mmc7.pdf]

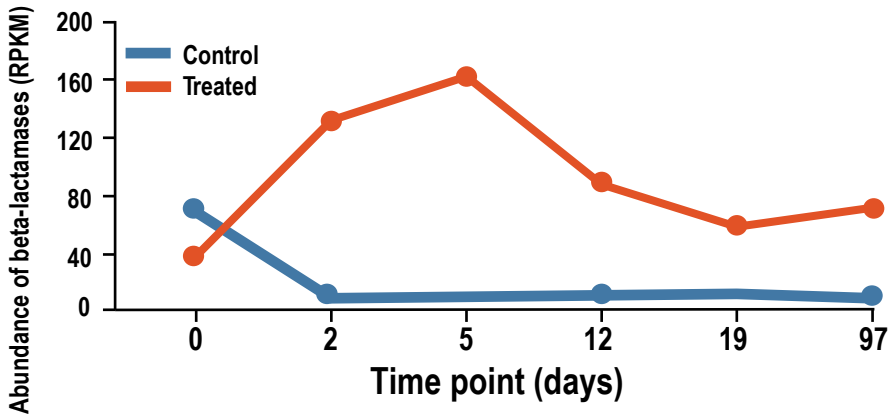

Supplement: Supplementary Figure S9 — Abundance variation for β-lactamases [file mmc9.pdf]

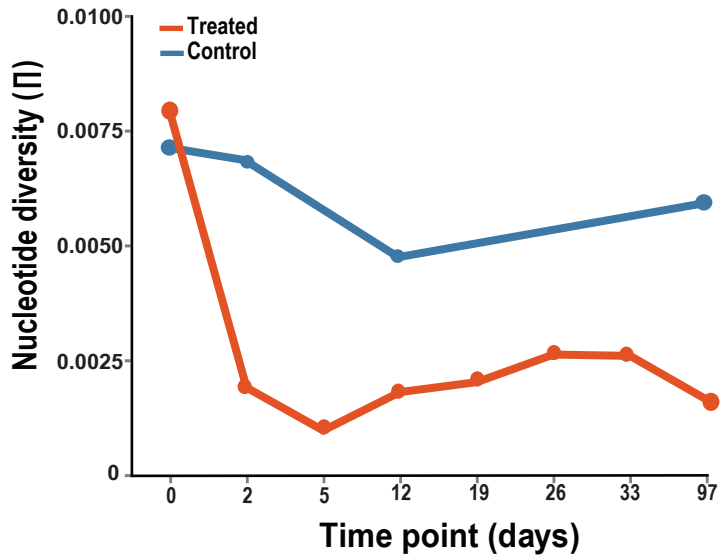

Supplement: Supplementary Figure S10 — Temporal variation of the nucleotide diversity in the predicted ARGs in both treated and control subject [file mmc10.pdf]

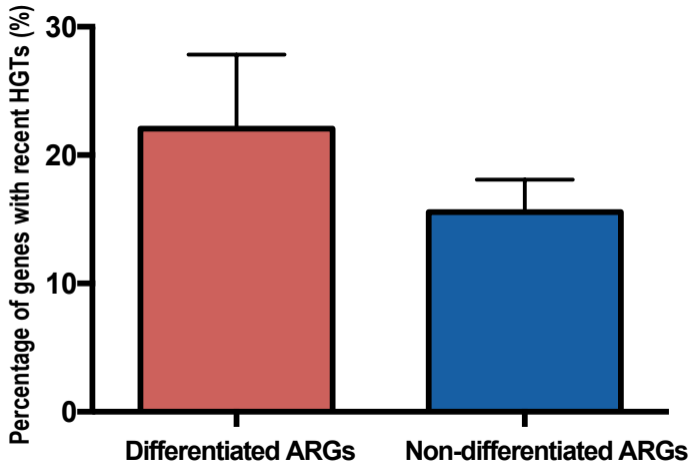

Supplement: Supplementary Figure S11 — The percentage of genes with recent HGT signatures based on public database [file mmc11.pdf]

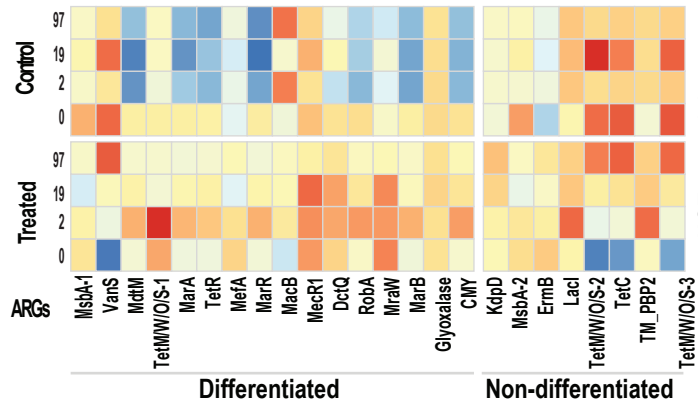

Differentiated  
Non-differentiated

HGT potential

1500  
750  
0

0 2 19 97

Time point (days)

1500  
750  
0

0 2 19 97

Abundance (RPKM)

25  
10  
1  
0.5

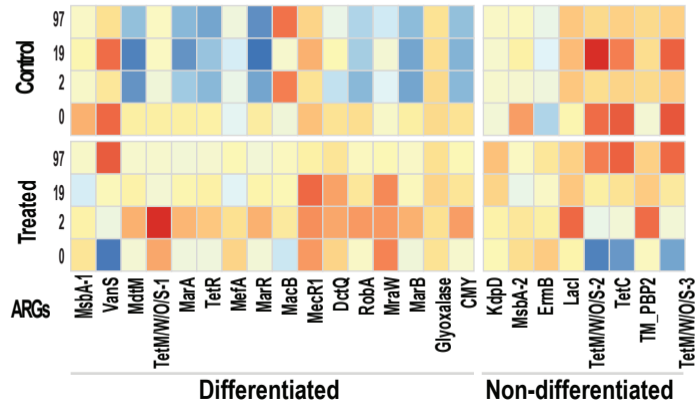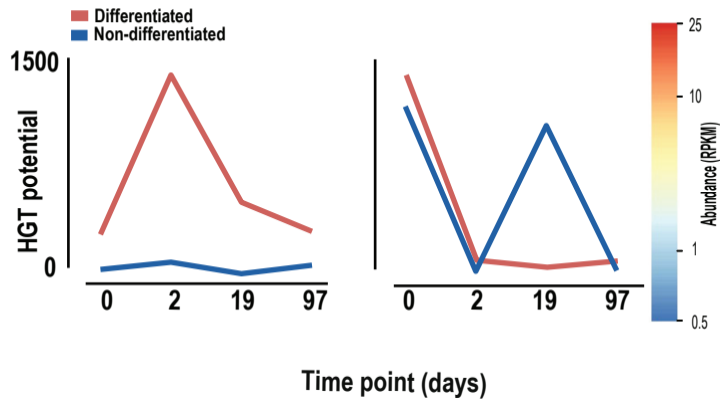

Supplement: Supplementary Figure S12 — Variation of the relative abundance and HGT potential of the functionally selected ARGs [file mmc12.pdf]
