# Supplementary material for: Antibiotic Treatment Drives the Diversification of the Human Gut Resistome
Source: Genomics Proteomics Bioinformatics. 2019 Apr 23;17(1):39–51. doi: 10.1016/j.gpb.2018.12.003 (PMC6520913; doi:10.1016/j.gpb.2018.12.003)
Supplement: Supplementary Figure S8 — Abundance variation for various types of antibiotic resistant genes in A. beta-lactam plates and B. non-beta-lactam plates [file mmc8.pdf]

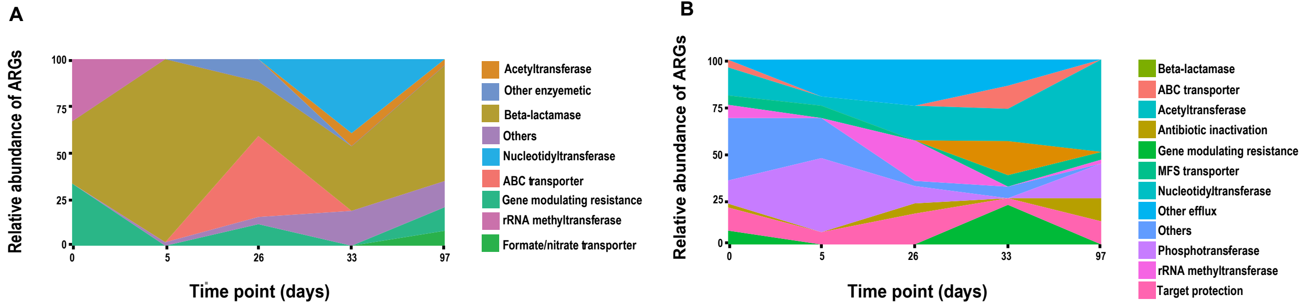
A
Relative abundance of ARGs
100
75
50
25
0
0
5
26
33
97
Time point (days)
Acetyltransferase
Other enzyemetic
Beta-lactamase
Others
Nucleotidyltransferase
ABC transporter
Gene modulating resistance
rRNA methyltransferase
Formate/nitrate transporter
B
Relative abundance of ARGs
100
75
50
25
0
0
5
26
33
97
Time point (days)
Beta-lactamase
ABC transporter
Acetyltransferase
Antibiotic inactivation
Gene modulating resistance
MFS transporter
Nucleotidyltransferase
Other efflux
Others
Phosphotransferase
rRNA methyltransferase
Target protection
