# Supplementary material for: Antibiotic Treatment Drives the Diversification of the Human Gut Resistome
Source: Genomics Proteomics Bioinformatics. 2019 Apr 23;17(1):39–51. doi: 10.1016/j.gpb.2018.12.003 (PMC6520913; doi:10.1016/j.gpb.2018.12.003)
Supplement: Supplementary Table S1 [file mmc13.docx]

**Table S1** **Information of 28 prevalent species used in the strain-level analysis**

| **ID of species group** | **Total pangenome genes** | **No. of ARGs** |
| --- | --- | --- |
| *Alistipes finegoldii* | 4572 | 6 |
| *Alistipes onderdonkii* | 4393 | 9 |
| *Alistipes putredinis* | 2372 | 3 |
| *Alistipes shahii* | 3299 | 5 |
| *Bacteroidales bacterium 58650* | 2848 | 5 |
| *Bacteroides caccae* | 5492 | 9 |
| *Bacteroides ovatus* | 26,316 | 42 |
| *Bacteroides rodentium* | 5135 | 9 |
| *Bacteroides thetaiotaomicron* | 12,013 | 26 |
| *Bacteroides uniformis* | 12,228 | 28 |
| *Bacteroides vulgatus* | 22,080 | 37 |
| *Bacteroides xylanisolvens* | 16,154 | 32 |
| *Barnesiella intestinihominis* | 3030 | 7 |
| *Bilophila wadsworthia* | 9156 | 23 |
| *Blautia wexlerae* | 8586 | 18 |
| *Burkholderiales bacterium* | 3287 | 8 |
| *Clostridiales bacterium* | 13,088 | 30 |
| *Eubacterium eligens* | 2665 | 10 |
| *Eubacterium rectale* | 7295 | 21 |
| *Faecalibacterium cf 62236* | 2825 | 7 |
| *Faecalibacterium prausnitzii* | 5170 | 14 |
| *Oscillibacter sp 60799* | 2830 | 6 |
| *Oscillospiraceae bacterium* | 5452 | 14 |
| *Parabacteroides distasonis* | 18,432 | 40 |
| *Parabacteroides merdae* | 6987 | 11 |
| *Ruminococcus bromii* | 2204 | 7 |
| *Subdoligranulum sp 62068* | 4483 | 16 |
