# Supplementary material for: Antibiotic Treatment Drives the Diversification of the Human Gut Resistome
Source: Genomics Proteomics Bioinformatics. 2019 Apr 23;17(1):39–51. doi: 10.1016/j.gpb.2018.12.003 (PMC6520913; doi:10.1016/j.gpb.2018.12.003)
Supplement: Supplementary Table S3 [file mmc15.docx]

**Table S3** **The fold-change (< 0.5) of genome-wide diversity during treatment for particular species**

| **Individual ID** | **Fold-change of genome-wide diversity during treatment** | **Treated (T) or control (C)** | **Species** |
| --- | --- | --- | --- |
| P18 | 0.34 | T | *Alistipes onderdonkii* |
| P10 | 0.35 | T | *Bacteroidales bacterium* |
| P10 | 0.3 | T | *Bacteroides caccae* |
| P19 | 0.33 | T | *Bacteroides uniformis* |
| P10 | 0.42 | T | *Bacteroides uniformis* |
| P17 | 0.41 | T | *Bacteroides uniformis* |
| P13 | 0.43 | T | *Bacteroides uniformis* |
| P17 | 0.4 | T | *Bacteroides vulgatus* |
| P10 | 0.26 | T | *Bacteroides vulgatus* |
| P10 | 0.47 | T | *Bacteroides xylanisolvens* |
| P15 | 0.49 | T | *Bacteroides xylanisolvens* |
| P22 | 0.38 | T | *Burkholderiales bacterium* |
| P10 | 0.25 | T | *Faecalibacterium prausnitzii* |
| P38 | 0.45 | C | *Faecalibacterium prausnitzii* |
| P9 | 0.48 | T | *Oscillospiraceae bacterium* |
| P10 | 0.49 | T | *Parabacteroides merdae* |
